# Association between periprosthetic tissue metal content, whole blood and synovial fluid metal ion levels and histopathological findings in patients with failed metal-on-metal hip replacement

**DOI:** 10.1371/journal.pone.0197614

**Published:** 2018-05-16

**Authors:** Lari Lehtovirta, Aleksi Reito, Jyrki Parkkinen, Sirpa Peräniemi, Jouko Vepsäläinen, Antti Eskelinen

**Affiliations:** 1 University of Tampere, Tampere, Finland; 2 Coxa Hospital for Joint Replacement, Tampere, Finland; 3 Fimlab Laboratories, Tampere, Finland; 4 University of Eastern Finland, Kuopio, Finland; University of Oulu, FINLAND

## Abstract

Adverse Reaction to Metal Debris (ARMD) is a major cause of implant failure leading to revision surgery in patients with metal-on-metal (MoM) hip arthroplasties. However, the pathogenesis and its association to implant wear are poorly understood and previous studies have yielded discrepant results. We sought to investigate the associations between histological findings, whole blood and synovial fluid metal ion concentrations and periprosthetic tissue metal concentrations in patients with MoM total hip replacements and hip resurfacings revised for ARMD. 107 hips in total were included in our study. Of these, 87 were total hip replacements and 20 were hip resurfacings, respectively. We found that whole blood, synovial fluid and periprosthetic tissue metal concentrations correlated poorly with histological findings. We suggest that the lack of a clear association between histological findings and wear measures in the present study as well as in previous studies is mostly influenced by variability in patient susceptibility. However, patients presenting with perivascular lymphocytic infiltration had lower chromium concentration in their periprosthetic tissues than patients with no perivascular lymphocytic infiltration. This may reflect the role of metal hypersensitivity in implant failure in these patients. Patients with total hip replacements evinced more necrosis and lymphocytic infiltration in their tissues than patients with hip resurfacings. This suggests that trunnion wear debris is more cytotoxic and/or immunogenic than bearing wear debris leading to higher failure rates seen in patients with total hip replacements.

## Introduction

Adverse Reaction to Metal Debris (ARMD) is a major cause of implant failure leading to revision surgery in patients with metal-on-metal (MoM) hip arthroplasties [1–5]. The term ARMD is an umbrella term describing periprosthetic soft-tissue reactions caused by metal wear debris that include metallosis, extra-articular pseudotumors (inflammatory, benign soft-tissue masses), overall inflammatory response of the tissue and variable amounts of necrosis seen as caseotic substance on macroscopic level. The term aseptic lymphocytic vasculitis-associated lesions (ALVAL) is more specific and was originally used to describe lymphocytic and necrotic tissue responses [6]. In recent literature terms ALVAL and ARMD have often been inappropriately used interchangeably [7]. The pathogenesis of these adverse reactions is poorly understood, but at least two different mechanisms have been suggested: 1. a non-specific, wear-particle induced cytotoxicity with foreign-body response [8,9] and 2. a specific, type IV hypersensitivity response involving recruitment of lymphocytes in the tissues around failed MoM hip replacements, manifesting as ALVAL [6,10].

Literature regarding implant wear and ARMD is inconclusive. Adverse reactions have been observed both in patients with high- and low-wearing hip replacements [11–15]. Several studies have investigated the associations between wear of the retrieved implants, or indirect markers of wear, and the histopathological findings of periprosthetic tissue taken at the revision surgery, but the results have been discrepant. Lymphocyte-dominated type IV response has been suggested as a cause of failure in patients with low-wearing implants [12,16–18] and cytotoxic response leading to macrophage recruitment in patients with high-wearing implants [8,12,16]. However, conflicting findings not supporting these hypotheses have been published as well [3,15,19–21]. To the best of our knowledge, there has only been one small-scale study that has directly measured the amount of metal in the periprosthetic tissues and analyzed its association with histopathological findings. In that study, Lohmann et. al found that high metal content in the periprosthetic tissue was associated with lymphocyte-dominated, and low metal content was associated with macrophage-dominated response [19]. These findings do not support the hypothesis of metal hypersensitivity as a cause of failure in low-wearing hips and foreign-body cytotoxic response in high-wearing hips.

Studies investigating wear, or indirect measures of wear, and histopathological findings have been inconclusive. The pathogenesis of ARMD and its association to implant wear is poorly understood as well as the potential difference between bearing surface wear debris and taper wear debris in the development of ARMD. Therefore, we aimed to investigate the associations between periprosthetic tissue metal content, whole blood (WB) metal ion concentrations, synovial fluid (SF) metal ion concentrations, and histopathological findings in patients with failed MoM total hip replacements compared to patients with failed MoM hip resurfacings.

## Materials and methods

We recruited a pilot patient for our study in June 2013 followed by the recruitment of consecutive patients between February 2014 and August 2016. In total, 134 hips with MoM implants were revised for ARMD at our institution during the period of recruitment. Of these, two hips were not included due to infection, two hips due to inadequate tissue sample and 23 hips were not included as they were operated on by surgeons who did not participate in recruitment and sample collection. Thus, 107 hips in total were included in our study. Of these, 87 were total hip replacements (THR) and 20 were hip resurfacings, respectively. Whole blood sample was available for 106 patients and synovial fluid sample for 90 patients. In addition to patients undergoing revision surgery, two further patients who had undergone primary hip arthroplasty and whose tissue samples had been retrieved from osteoarthritic synovium were recruited as controls for tissue metal analysis. Surgery was performed by or under the direct supervision of 14 senior orthopedic surgeons. Patient demographics and revised components are presented in detail in Table 1. All patients gave written informed consent to participate in this study, and the study was also approved by the institutional ethical committee (Ethics Committee of Pirkanmaa Hospital District, decision R11196).

**Table 1:** Patient demographics and implant designs.

**Patient demographics** Mean age at the time of revision 66.8 years (SD 7.5 years) Mean follow-up time between index and revision operation 7.1 years (SD 2.5 years) Gender ratio: 42 females (42%) and 57 males (58%) **Revised implants**		
**Total hip replacements**		**Amount**
*Femoral component*	*Acetabular component*	
DePuy Summit	DePuy ASR	32
DePuy Summit	DePuy Pinnacle	10
Biomet Bimetric	Biomet M2A38	10
Biomet Bimetric	Biomet ReCap	5
DePuy Corail	DePuy ASR	4
Smith-Nephew Synergy	Smith-Nephew R3	4
Zimmer ZMR	Zimmer Durom	2
Zimmer M/L Taper	Zimmer Durom	2
Wright Medical Profemur	Wright Medical Conserve Plus	2
Other	Other	16
		Total = 87
**Hip resurfacings**		
DePuy ASR		9
Smith-Nephew BHR		6
Zimmer Durom		2
Biomet ReCap		2
Smith-Nephew BHR—TM Revision shell		1
		Total = 20

Revision surgery was considered, as previously described [2,22–24], if 1) a clear pseudotumour (Imperial class 2A, 2B or 3) [25] was observed on cross-sectional imaging regardless of symptoms or whole blood metal ion levels; or 2) the patient had elevated whole blood metal ion levels and hip symptoms despite normal findings in cross-sectional imaging; or 3) the patient had a continuously symptomatic hip or progressive symptoms regardless of imaging findings or metal ion levels. Symptoms included hip pain, discomfort, sense of instability, and/or impaired function of the hip and sounds from the hip (clacking, squeaking). Whole blood metal ion levels were regarded as being elevated if either chromium or cobalt exceeded 5 ppb. Postoperatively, failure was classified as being due to ARMD and included in our study if the following criteria were met: 1) there was presence of metallosis or macroscopic synovitis in the joint; and/or 2) a pseudotumor was found during revision; and/or 3) a moderate to high number of perivascular lymphocytes along with tissue necrosis and/or fibrin deposition was seen in the histopathologic sample; and 4) perioperatively there was no evidence of component loosening or periprosthetic fracture. In addition, infection was ruled out by obtaining multiple (at least five) bacterial cultures during revision surgery.

### Metal analysis of the periprosthetic tissue

During every revision surgical procedure, samples of the inflamed synovia and/or pseudotumor were obtained for both histopathological and metal content analysis. For metal content analysis, a subsample (approx. 0.3 g) was cut from the tissue sample, weighed, and transferred into a teflon vessel. Samples were first decomposed with 5 ml suprapur HNO3 (Merck) by microwave digestion technique using a CEM MDS-2000 Microwave System (CEM corporation, Matthews, NC, USA) and then diluted to 10 ml with Milli Q-water. The digests were analyzed for Al, Cr, Co, Ti, Mo, and V with a Inductively Coupled Plasma Optical Emission Spectrometer. Thermo Electron iCAP 6600 Duo View equipped with Cetac ASX-520Hs and autosampler was used (Thermo Fisher Scientific, Waltham, MA, USA). Detection limits for Al, Cr, Co, Ti, Mo, and V were 9.0, 0.2, 0.2, 3.0, 0.2 and 3.0 μg/g, respectively. NIST SRM 1576b (Bovine liver) was used as certified reference material to ensure the performance of analytical procedure for tissue samples.

### Histopathological analysis of the periprosthetic tissue

For histopathological analysis, each tissue sample was formalin fixed and embedded in paraffin. Several 10 μm microtome sections were made. Standard hematoxylin and eosin staining was used. The sections were examined histologically under normal light with a Nikon Eclipse 50i (Nikon Corporation, Shinagawa, Tokyo, Japan). The samples were graded by a senior musculoskeletal pathologist (JP) using grading described by Natu et. al [10]. The grading consisted of following parameters: 1) lymphocyte cuff thickness, 2) whether diffuse lymphocytic infiltration was present, 3) presence of germinal centers, 4) histiocyte sheet thickness, 5) metal particle load within histiocytes, 6) Grade of tissue necrosis, 7) presence of plasma cells and 8) presence of granulomas. Lymphocytic cuff thickness was calculated using a 1mm eyepiece graticule. Calculations were done using 10x magnification. An average of five measurements was taken and graded as 0–3 (absent, 0.25 mm, 0.25–0.75 mm, >0.75 mm). Macrophage sheet thickness was also calculated using a graticule and graded 0–3 (absent, <1 mm, 1–2 mm, >2mm). Metal particle load within macrophages was graded as 0–4 as done in the assessment of iron decomposition in liver cells [26,27]. The extent of overall tissue necrosis in a sample was graded based on the surface necrosis typing according to Davies et al. [28]. Type 1 surface contains intact synovial epithelium. Type 2 surface shows loss of synovial epithelial cells without fibrin deposition. In type 3 surface there is fibrin deposition and in type 4 surface there is extensive necrosis and loss of architecture. The extent of type 4 surface necrosis was used to grade the overall tissue necrosis in a given sample, as described by Natu et al. [10]. In grade 4 necrosis, more than 75% of the tissue sample showed type 4 surface necrosis. In grade 3 necrosis, between 25% and 75% showed type 4 surface necrosis. In grade 2 necrosis either less than 25% of the tissue showed type 4 surface necrosis or the tissue showed type 3 surface. In grade 1 necrosis, the sample consisted of type 2 surface.

### Whole blood and synovial fluid metal analysis

Since January 2012, WB metal ion (Co and Cr) concentrations have been routinely measured as a part of the systematic follow-up program for patients with MoM hip replacements at our institution. All patients underwent WB analysis of Co/Cr following sampling from the antecubital vein using a 21-gauge needle connected to a Vacutainer system (Becton, Dickinson and Company, Franklin Lakes, NJ, USA) and trace-element blood tubes containing sodium ethylenediaminetetraacetic acid (EDTA). Standard operating procedures were established at the Finnish Institute for Occupational Health for Co and Cr measurement using dynamic reaction cell inductively coupled plasma (quadripole) mass spectrometry (Agilent 7500 cx, Agilent Technologies, Santa Clara, CA, USA). The laboratory technicians were blinded to all clinical outcomes. The samples were preserved in +6 °C to +8 °C prior to analysis.

Since October 2011, our MoM hip revision protocol has involved perioperative SF aspiration, which is always taken before opening the deep fascia using a standard 18- to 20-gauge needle connected to a Vacutainer system (Becton, Dickinson and Company, Franklin Lakes, New Jersey) and trace element tubes containing sodium EDTA. Similar procedures were used for SF metal ion concentration measurement as described above for WB.

### Statistical analysis

Spearman rank correlation was used to study the associations between tissue metal contents, WB and SF metal ion concentrations, and histopathological measures due to these variables being non-normally distributed. Medians were calculated for the tissue metal contents and the histopathological measures. Mann-Whitney U-test was used for comparing medians. When analyzing the correlation between WB metal ion concentrations and other factors, we only included patients with unilateral hip replacements (69 patients with total hip replacement and 9 patients with hip resurfacing) to avoid the confounding effect of metal ions being released to the blood from a second source. The internal validity of our study was investigated by correlating the microscopically visible metal particles with the tissue metal content. We should observe significant association to have a valid method for metal content assessment. The threshold for statistical significance was set to 0.05. The analyses were conducted using IBM SPSS version 21.

## Results

Chromium had the highest concentration of all metals in the periprosthetic tissue in both the HR and THR groups (Table 2). In whole blood, however, cobalt ions were present in higher concentrations than chromium ions (Table 3). Titanium was elevated above the detection limit in nine patients with hip resurfacing and in 29 patients with THR. The concentrations for aluminum and vanadium did not reach the detection limit in any of the patients and were thus omitted from the analyses. There were no statistically significant differences in periprosthetic tissue metal concentrations between THR and hip resurfacing groups (Table 2). In whole blood, median cobalt concentration was approximately twice as high in the THR group compared to hip resurfacing group (Table 3). There was no difference in whole blood chromium ion concentration between the groups (Table 3). In the tissue samples of the two control patients, only the concentration of chromium exceeded the detection limit (0.3 μg/g and 0.5 μg/g, respectively).

**Table 2:** Median values with respected p-values and ranges for periprosthetic tissue metal concentrations in patients with total hip replacements (n = 87) and hip resurfacings (n = 20).

Metal	Total hip replacement		Hip resurfacing		
	Median concentration in tissue (μg/g)	Range (μg/g)	Median concentration in tissue (μg/g)	Range (μg/g)	P-value
Chromium	39.2	0.4–1955.0	43.8	0.6–922.1	0.60
Cobalt	6.4	0.2–262.0	3.2	0.2–248.8	0.189
Molybdenium	1.8	0.2–174.6	0.5	0.2–32.4	0.080
Titanium	5.8	3.0–118.9	4.9	4.9–25.3	0.10

**Table 3:** Median values, respected p-values and ranges for whole blood metal ion concentrations in patients with unilateral total hip replacement (n = 69) or hip resurfacing (n = 13) patients.

Metal	Total hip replacement		Hip resurfacing		
	Median concentration in whole blood (μg/l)	Range (μg/l)	Median concentration in whole blood (μg/l)	Range (μg/l)	P-value
Chromium	3.7	0.4–29.9	3.9	1.5–7.2	0.60
Cobalt	11.0	0.6–108.5	3.9	1.5–16.2	0.001

Lymphocyte cuff thickness score was higher in patients with THRs versus hip resurfacing (Table 4) and the difference was statistically significant (p = 0.011). Macrophage sheet thickness between hip resurfacing and THR groups did not differ significantly (Table 4, p = 0.65). The grade of tissue necrosis was higher in the THR group compared to hip resurfacing group (Table 4, p < 0.0001).

**Table 4:** Lymphocyte cuff thickness, macrophage sheet thickness and grade of necrosis in total hip replacement group (n = 87) and hip resurfacing group (n = 20).

		Total hip replacement	Hip resurfacing	P-value
**Lymphocyte cuff thickness**	**0 (absent)**	33 (37.9%)	15 (75%)	
	**1 (0–0.25mm)**	41 (47.1%)	4 (20%)	
	**2 (>0.25mm)**	13 (14.9%)	1 (5.0%)	
				0.011
**Macrophage sheet thickness**	**0 (absent)**	1 (1%)	0 (0%)	
	**1 (<1mm)**	68 (78.2%)	18 (90%)	
	**2 (1-2mm)**	16 (18.4%)	2 (10%)	
	**3 (>2mm)**	2 (2.3%)	0 (0%)	0.65
**Grade of necrosis**	**1**	3 (3.4%)	8 (40%)	
	**2**	19 (21.8%)	4 (20%)	
	**3**	12 (13.8%)	1 (5%)	
	**4**	53 (60.9%)	7 (35%)	
				<0.001

Correlations between histological variables, periprosthetic tissue metal concentrations, whole blood metal ion levels and synovial fluid metal ion levels are presented in Table 5. Of all the variables, only metal particle load within macrophages had statistically significant but weak correlations with metal ion levels in tissues and whole blood in the THR group. In the resurfacing group, only the synovial fluid chromium and metal particle load had a statistically significant correlation. Correlation between lymphocyte cuff thickness and periprosthetic tissue chromium concentration trended towards significance in the THR group (ρ = -0.20, p = 0.063).

**Table 5:** Correlations between histological findings, periprosthetic tissue metal concentrations, whole blood metal ion levels (WB) and and synovial fluid (SF) metal ion levels in total hip replacement group (n = 87) and hip resurfacing group (n = 20). Cells containing statistically significant values are colored in gray.

	Total hip replacement				Hip resurfacing			
	*Lymphocytic cuffing*	*Macrophage sheet thickness*	*Grade of necrosis*	*Metal particle load*	*Lymphocytic cuffing*	*Macrophage sheet thickness*	*Grade of necrosis*	*Metal particle load*
*Tissue chromium*	rho = -0.20 p = 0.063	rho = 0.022 p = 0.84	rho = -0.13 p = 0.22	rho = 0.34 p < 0.01	rho = -0.36 p = 0.12	rho = 0.12 p = 0.63	rho = -0.28 p = 0.23	rho = 0.29 p = 0.22
*Tissue cobalt*	rho = -0.072 p = 0.51	rho = 0.031 p = 0.78	rho = -0.001 p = 0.99	rho = 0.30 p < 0.01	rho = -0.35 p = 0.13	rho = 0.09 p = 0.72	rho = -0.28 p = 0.23	rho = 0.26 p = 0.27
*Tissue molybdenium*	rho = -0.071 p = 0.514	rho = 0.060 p = 0.584	rho = -0.069 p = 0.53	rho = 0.25 p = 0.019	rho = -0.29 p = 0.21	rho = 0.03 p = 0.90	rho = -0.34 p = 0.15	rho = 0.30 p = 0.20
*Tissue titanium*	rho = -0.017 p = 0.88	rho = -0.036 p = 0.74	rho = -0.035 p = 0.74	rho = 0.11 p = 0.30	rho = -0.16 p = 0.51	rho = -0.030 p = 0.60	rho = -0.13 p = 0.58	rho = -0.077 p = 0.75
*WB Cr*	rho = -0.092 p = 0.45	rho = 0.043 p = 0.73	rho = 0.011 p = 0.92	rho = 0.21 p = 0.085	rho = -0.34 p = 0.25	rho = 0.29 p = 0.35	rho = 0.14 p = 0.66	rho = 0.32 p = 0.29
*WB Co*	rho = -0.088 p = 0.47	rho = -0.053 p = 0.67	rho = 0.10 p = 0.41	rho = 0.39 p < 0.01	rho = -0.11 p = 0.72	rho = 0.29 p = 0.35	rho = 0.33 p = 0.27	rho = 0.53 p = 0.067
*SF Cr*	rho = -0.096 p = 0.39	rho = 0.020 p = 0.86	rho = -0.077 p = 0.49	rho = 0.15 p = 0.18	rho = -0.46 p = 0.22	rho = 0.00 p = 1.00	rho = 0.11 p = 0.78	rho = 0.77 p = 0.016
*SF Co*	rho = 0.053 p = 0.64	rho = 0.12 p = 0.30	rho = 0.17 p = 0.12	rho = 0.17 p = 0.14	rho = -0.43 p = 0.25	rho = 0.21 p = 0.59	rho = 0.19 p = 0.62	rho = 0.59 p = 0.096

In the THR group in tissues with no lymphocyte infiltration at all, median chromium concentration was higher than in tissues with lymphocyte infiltration present (Table 6). In regard to cobalt and molybdenium there were no statistically significant differences. In the hip resurfacing group, there was a trend towards lower concentrations of chromium and cobalt in those tissues with lymphocytes present but these differences did not reach statistical significance (p = 0.11 and p = 0.12, respectively) (Table 7).

**Table 6:** Median metal concentration in tissues with lymphocytes present and tissues with no lymphocytes present in the total hip replacement group (n = 87).

Median concentration in tissue (μg/g)	Lymphocytes present	No lymphocytes present	P-value
Chromium	30.1	67.4	0.045
Cobalt	6.4	6.1	0.43
Molybdenium	1.7	1.8	0.38

**Table 7:** Median metal concentration in tissues with lymphocytes present and tissues with no lymphocytes present in the hip resurfacing group (n = 20).

Median concentration in tissue (μg/g)	Lymphocytes present	No lymphocytes present	P-value
Chromium	8.0	79.3	0.11
Cobalt	1.2	4.2	0.12
Molybdenium	0.3	0.69	0.20

Periprosthetic tissue chromium and cobalt concentrations correlated weakly with whole blood and synovial fluid chromium and cobalt concentrations in THR group (Table 8). In resurfacing group, only synovial fluid cobalt concentration reached statistically significant correlation with periprosthetic tissue cobalt concentration (Table 8).

**Table 8:** Spearman rho correlation coefficients between tissue metal concentrations, whole blood (WB) and synovial fluid (SF) metal ion concentrations in total hip replacement (n = 87) and hip resurfacing (n = 20) groups.

	Total hip replacement		Hip resurfacing	
	*Tissue chromium*	*Tissue cobalt*	*Tissue chromium*	*Tissue cobalt*
*WB chromium*	rho = 0.32, p<0.01		rho = 0.48, p = 0.10	
*WB cobalt*		rho = 0.31, p<0.01		rho = 0.24, p = 0.43
*SF chromium*	rho = 0.29, p<0.01		rho = 0.63, p = 0.067	
*SF cobalt*		rho = 0.34, p<0.01		rho = 0.70, p = 0.035

## Discussion

In the present study, we analyzed periprosthetic tissue metal concentrations, whole blood metal ion concentrations, synovial fluid metal ion concentrations and performed thorough histological analysis of periprosthetic tissue using grading described by Natu et al. [10]. Patients with THR evinced significantly higher amounts of lymphocytes and necrosis in their tissues compared to patients with hip resurfacings despite similar metal concentrations in periprosthetic tissues. Also, patients with total hip replacements had higher whole blood cobalt ion concentrations compared to patients with hip resurfacings. Histological findings that reflect the inflammatory response and necrosis of the tissues correlated poorly with any of the metal ion measurements. However, periprosthetic tissues with lymphocytic infiltration present had lower amounts of chromium than tissues with no lymphocytic infiltration present.

This study is not without limitations. Firstly, although we performed consecutive recruitment of patients, not all patients who underwent surgery because of ARMD during the recruitment period were included in our study due to some surgeons not participating in the recruitment and some patients being excluded due to infection or inadequate tissue sample. Thus, our series of patients is not completely consecutive. Secondly, we performed semiquantitative histological grading of the samples using grading described by Natu et al. [10]. Grading was done by one observer only. Thirdly, tissue samples used for metal ion measurement were rather small (approx. 0.3g) and may not have completely reflected the average metal concentration of the whole synovium. Also, we were not able to differentiate between metal ions, metals bound to proteins and larger metal particles in the measurement of tissue metal content.

Chromium was the most prominent metal in the periprosthetic tissue in both study groups, which is in line with previous research [19,29–32]. Median concentrations of chromium in the periprosthetic tissue exceeded those of cobalt by more than six-fold in both study groups. On the contrary, in whole blood cobalt ion concentration was higher than that of chromium in the total hip replacement group. Chromium is known to accumulate in the tissues to a high degree while cobalt ions are rapidly transported to the blood and eliminated in the urine [33,34] which explains why chromium concentration is higher than cobalt in periprosthetic tissues and cobalt concentration higher than chromium in whole blood. However, in the hip resurfacing group the cobalt and chromium concentrations in whole blood were similar. This could be due to the small sample size of the hip resurfacing group. Cobalt concentration in whole blood was approximately twice as high in THR group compared to hip resurfacing group while chromium concentrations did not differ between implant groups. Similar findings have been published [35–37]. The excess cobalt in patients with a THR is likely due to material loss at the trunnion surface [38,39]. Periprosthetic metal concentrations correlated poorly with whole blood and synovial fluid metal ion concentrations. The only exception was the good correlation between synovial fluid and periprosthetic tissue cobalt concentrations. We suggest that the overall poor correlations are due to tissues reflecting the accumulated metal load while whole blood and synovial fluid reflect the amount of wear that has been generated more recently. Also, in whole blood and synovial fluid only metal ions are measured whereas in tissues all forms of metal, including particles, ions and metallo-organic complexes, are included in the total amount of metal. Titanium was elevated in 29 patients implying its release from the stem, acetabular cup, or head-neck trunnion. Since this elevation was also seen in patients with hip resurfacings, release from the outer surface of acetabular cup seems probable. Vendittoli et al. found that serum titanium concentrations were indeed higher in hip resurfacings than THRs [40]. In the present study, we did not observe a statistically significant difference in titanium levels between THR and hip resurfacing groups.

We found that periprosthetic tissues retrieved from patients with total hip replacements evinced more severe necrosis and more lymphocytes compared to tissues retrieved from patients with hip resurfacings. Taper wear debris has been suggested to be more immunogenic and cytotoxic than bearing wear debris [41,42]. Xia et al. compared tissues from patients with dual-modular non-MoM implants, MoM THR and MoM hip resurfacings [42]. In dual-modular implants there are two modular junctions which serve as a source of trunnion wear, whereas in THR there is one modular junction and in hip resurfacing there are no modular junctions at all and all wear debris originates from the bearing surfaces. Xia et al. found that tissues from patients with dual-modular implants had highest amounts of lymphocytes and tissue destruction, tissues from THR patients having lower amounts and ultimately tissues from hip resurfacing patients having the lowest amounts. This was despite the fact that tissues from patients with dual-modular non-mom implants had de facto lowest amount of metal debris. Also, patients with dual-modular implants had shortest time to failure. The authors concluded that trunnion wear is likely more immunogenic and cytotoxic than bearing wear debris, leading to rapid failure. Our results support these findings and suggest that taper wear may cause more tissue destruction than bearing wear manifesting as substantially higher failure rates for THRs than hip resurfacings despite similar amounts of metals in periprosthetic tissues.

In the present study, periprosthetic tissue, whole blood and synovial fluid metal concentrations had poor correlations with histological findings. Several retrieval studies have been conducted to study the relationship between implant wear and histopathological findings. Campbell et al. investigated the amount of implant wear and type of tissue response in patients with failed MoM hips and found that low wear was associated with a hypersensitivity type lymphocytic response [12]. Conversely, high component wear was associated with a macrophage-dominated response suggesting non-specific wear-related cytotoxicity. Slightly differently, Grammatopoulos et al. found that implant wear was associated with the number of macrophages but not with the number of lymphocytes [8]. In their study, all patients with a pseudotumor and a low-wearing implant had a high ALVAL score suggesting a hypersensitivity response. However, most pseudotumors were associated with highly worn prostheses. A recent study by Paukkeri et al. found that whole blood chromium and cobalt ion correlations, indirect markers of wear, were higher in patients with macrophage-dominated response and lower in patients with lymphocyte-dominated response. On the contrary, Liow et al. found no correlation between whole blood metal ion levels and histological findings in periprosthetic tissue. To the best of our knowledge, only one previous study has investigated the periprosthetic metal content in relation to histopathological findings in patients with failed MoM hip arthroplasties [19]. Lohmann et al. found that high periprosthetic tissue metal content (chromium, cobalt and nickel combined and separately) was associated with a lymphocyte-dominated response and low metal content with a macrophage-dominated response. We would like to address some weaknesses in the study which may have affected the outcome. Firstly, the small number of cases in that study is likely to be a limiting factor. There were only five patients in the macrophage-dominated group and 22 patients in the lymphocyte-dominated group. The high incidence for the lymphocyte-dominated response compared to the macrophage-dominated response is neither supported by previous studies [8,10,12] nor the results of our study. Furthermore, mean values for tissue metal concentration were calculated and compared between the two groups. With nonparametric variables, this is not a valid statistical method. In conclusion, literature regarding the association between histological findings and wear or indirect measures of wear is very discrepant. A recent review suggested that periprosthetic tissue metal concentrations may correlate more accurately with the histology than serum metal ion levels [7]. Our results do not support that hypothesis. We found that tissue metal concentrations as well as whole blood and synovial fluid metal ion concentrations had poor correlations with histological findings. However, tissues with lymphocytic infiltration had lower amounts of chromium compared to tissues with no lymphocytic infiltration. This finding alone supports the hypothesis of hypersensitivity as a cause of failure in patients with low-wearing MoM hip implants. However, there was no correlation between the amount of lymphocytes and periprosthetic chromium concentration, which makes it difficult to draw conclusions in light of the overall results.

Associations between histological findings and wear or indirect measures of wear has been inconsistent and weak in previous studies as well as the present study. In the literature, the histopathology of ARMD tissues has mainly been categorized into a wear-related foreign-body response or a supposedly hypersensitivity-related lymphocyte-type response or a mix of both. It is possible and probable that some patients have both high-wearing implants and an underlying hypersensitivity-type response that would have evoked even in the presence of a low-wearing implant. This combination may result in a mixed-type tissue response that has the characteristics of both wear-related innate immune responses and hypersensitivity-related adaptive tissue responses and therefore makes it difficult to distinguish between the two based on the tissue metal content or some other measure of wear. This may also explain why we did not find correlation between tissue metal concentrations and lymphocytes, but did find a difference in concentration of metals between those with no lymphocytes versus those with lymphocytes present. It is possible that the differences between different lymphocyte scores are too subtle and vulnerable to error for a statistically significant correlation to be detected between these scores and metal concentrations in tissues. In contrast, dividing the patients in two groups: those with perivascular lymphocytes and those without, may thus reflect the association between inflammatory response and tissue metal concentration more clearly. Also, trunnion wear from THR appears to elicit different tissue responses than bearing wear, which makes comparison between studies difficult. In numerous previous studies, patient susceptibility has been suggested as an important factor contributing to the development of ARMD [12,14,15,17]. Patient susceptibility means that patients can elicit different types of responses to the metal debris at different levels of metal load in their tissues. We suggest that variability in the threshold level of metal debris needed to cause significant tissue responses explains the weakness and inconsistency between histological findings and wear measurements. The role of patient susceptibility in the pathogenesis of ARMD warrants further research.

## Conclusions

In conclusion, periprosthetic tissue metal concentrations had poor correlation with histological findings or metal ion levels in whole blood and synovial fluid. We suggest that this is mostly due to variation in patient susceptibility manifesting as individually different levels of reactivity to metal debris. Despite the similar metal concentrations in periprosthetic tissues, patients with THR evinced more lymphocytes and necrosis in their tissues compared to patients with hip resurfacings. We suggest that taper wear debris from THR is more immunogenic or cytotoxic compared to bearing surface wear debris, leading to higher failure rates in patients with THRs compared to hip resurfacings. In THR, tissues with lymphocytic infiltration had lower amounts of chromium than tissues with no lymphocytic infiltration. Similar trend was observed in hip resurfacings, but this did not reach statistical significance. These findings alone support the hypothesis of metal hypersensitivity as a cause of failure in a subgroup of patients with low-wearing hip implants. Interestingly, however, we did not observe correlation between lymphocyte scores and periprosthetic tissue chromium concentrations. Thus, it is difficult to draw solid conclusions regarding the role of metal hypersensitivity as a cause of failure in patients with low-wearing hip implants.
